# Kaleidoscope in the Mind: Charles Bonnet Syndrome

**DOI:** 10.1192/bjo.2025.10756

**Published:** 2025-06-20

**Authors:** Joel Philip, Feena Sebastian

**Affiliations:** Essex Partnership University NHS Foundation Trust, Wickford, United Kingdom

## Abstract

**Aims:** Charles Bonnet syndrome (CBS) is a phenomenon characterized by complex visual hallucinations in visually challenged patients. CBS may go unrecognized and be misdiagnosed as early dementia or psychosis.

**Methods:** We present the case of an 83-year-old Caucasian gentleman ‘X’ who was referred to the older adult home treatment team.

X reported that his problems began when some men turned up at his door a few weeks earlier, regarding an application he had made to the council for solar panels. X suffered from a complex delusional framework, believing that these men have stayed around in his home and are living in his loft. He believed that there are microphones in his bedroom and that his conversations are being transmitted to malicious people who work for the council.

X reported distressing visual hallucinations of men in his kitchen threatening him with guns. Other hallucinations included his ex-partner engaging in sexual activities with the aforementioned men, which led him to believe that she has been forced into the sex trade. He reported seeing distorted faces in his bathroom mirror, bodies floating up against the ceiling, and children with metallic prostheses for limbs. X recently received a diagnosis of a mild cognitive impairment following a referral made by the GP to the memory clinic. Prior to this, he did not have a history of psychiatric or neurological illness.

Interestingly, X had previously undergone excision of his right eyeball for the treatment of cancer, and has a skin graft at the site. He has only partial vision in his remaining eye due to macular degeneration.

**Results:** Three criteria should be fulfilled to diagnose CBS; namely, visual loss, clearly formed recurrent visual hallucinations, and insight into the unreal nature of the hallucinations.

In our case study, X fulfilled the first 2 criteria, but was unable to appreciate that the images that he sees are not real. We surmise that X may initially have understood that his hallucinations are not real, but these hallucinations gradually became amalgamated with his delusional belief systems, leading to a loss of insight.

**Conclusion:** CBS should be considered as a diagnostic possibility in persons with visual impairment presenting with visual hallucinations, and differentiated from psychotic and neurological disorders such as dementia, delirium and late-onset psychosis.

